# Metachronous Cutaneous Melanoma Metastases of the Left Anterior Paranasal Sinuses and the Right Nasopharynx That Were Treated Surgically: A Case Report With Literature Review

**DOI:** 10.7759/cureus.71792

**Published:** 2024-10-18

**Authors:** Aris I Giotakis, Ourania Natsiopoulou, Eleni Bouka, Dimitra Riga, Spyridon Lygeros, Evangelos Giotakis

**Affiliations:** 1 First Department of Otorhinolaryngology, Hippocration General Hospital Athens, Medical University of Athens, National and Kapodistrian University of Athens, Athens, GRC; 2 Department of Pathology, Hippocration General Hospital, Athens, Athens, GRC; 3 Department of Otolaryngology, University of Patras, Patras, GRC

**Keywords:** endoscopic surgical procedure, malignant melanoma metastasis, nasopharynx and the oral cavity. furthermore, nose surgery, paranasal sinuses pathology

## Abstract

Metastases of cutaneous melanoma to the paranasal sinuses and nasopharynx are considered rare. We reported the case of a patient with cutaneous melanoma metastasizing to the left anterior infundibulum and seven months later to the right nasopharynx that was successfully treated by endonasal endoscopic surgery as a single treatment modality. No adjuvant radiotherapy was performed. We described all known cases of metastatic cutaneous melanomas to the paranasal sinuses and nasopharynx. In cases of neither lung involvement nor open foramen ovale, the metastatic route might be the vertebral venous system. The histopathologic differentiation between primary mucosal and metastatic cutaneous melanoma remains critical. Adequate surgical treatment is crucial. More data are needed to further understand the role of adjuvant radiotherapy. Molecular markers, such as KIT and BRAF mutations, should be examined.

## Introduction

Metastases of cutaneous malignant melanoma to the paranasal sinuses are extremely rare [1,2]. Hada was the first to report a case in 1914 [3]. Since then, 12 cases have been described [4-12]. Three out of 12 patients survived and underwent surgery with adjuvant radiotherapy [5,7,10,11]. Metastases to the nasopharynx are also extremely rare, with 12 described cases [6,9,13]. Most metastases proved fatal, regardless of the treatment modality.

Here, we present the case of a patient with metachronous metastases of a cutaneous melanoma to the left anterior infundibulum and the right nasopharynx that was successfully treated by endonasal endoscopic surgery as a single treatment modality. The institutional review board approved this case report (68/26-09-2024). Informed written consent was obtained from the patient.

## Case presentation

A 70-year-old man was treated in August 2008 with surgical amputation of the left first toe due to a cutaneous nodular melanoma. Clark scale was IV, and Breslow thickness was 1.48 mm. Pathologic lymph nodes were not found at the time of diagnosis. Details about the BRAF mutation status were not available. Since then, the patient has undergone multiple treatments, such as lymph node dissection, chemotherapy, and immunotherapy, due to regional recurrences and in-transit metastatic disease.

**Figure 1:**
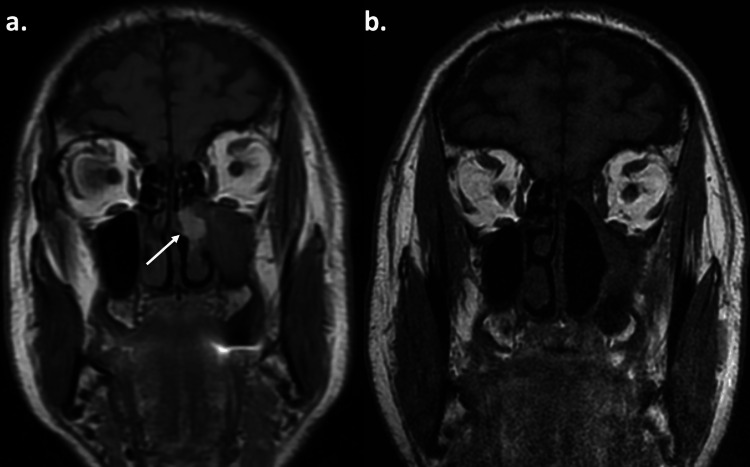
Before and after excision of the left paranasal sinus metastasis (a) Magnetic resonance imaging (MRI) of the head (T1-sequence), revealing a tumor (pointed by the white arrow) in the left infundibulum. (b) Postoperative MRI of the head (T1-sequence) showing the left median maxillectomy, ethmoidectomy and resection of the middle turbinate.

In November 2017, the patient reported left nasal obstruction, midfacial pressure, and unilateral epistaxis. Nasal endoscopy revealed a brown-black tumor of the left nasal infundibulum. Computed tomography of the head and neck with contrast showed full opacification of the left infundibulum and partial opacification of the left maxillary- and anterior ethmoid sinus. Magnetic resonance imaging (MRI) of the head and neck revealed a tumor of the left infundibulum infiltrating the middle and inferior turbinates (Figure 1a). The tumor extended to the superior, posterior, and middle parts of the maxillary sinus and to the inferior part of the anterior ethmoid sinus. A biopsy of the tumor revealed melanoma metastasis.

The multidisciplinary tumor board (MDT) suggested surgical treatment. In December 2017, endonasal endoscopic surgery was performed. Here, a left medial maxillectomy, frontoethmoidectomy, and resection of the middle turbinate were performed, accompanied by drilling the left maxillary sinus walls. No complications were observed. The patient was released three days later. Histologic examination confirmed the initial biopsy. MDT recommended no adjuvant treatment. The patient was free of nasal disease in the follow-up endoscopic examinations.

**Figure 2:**
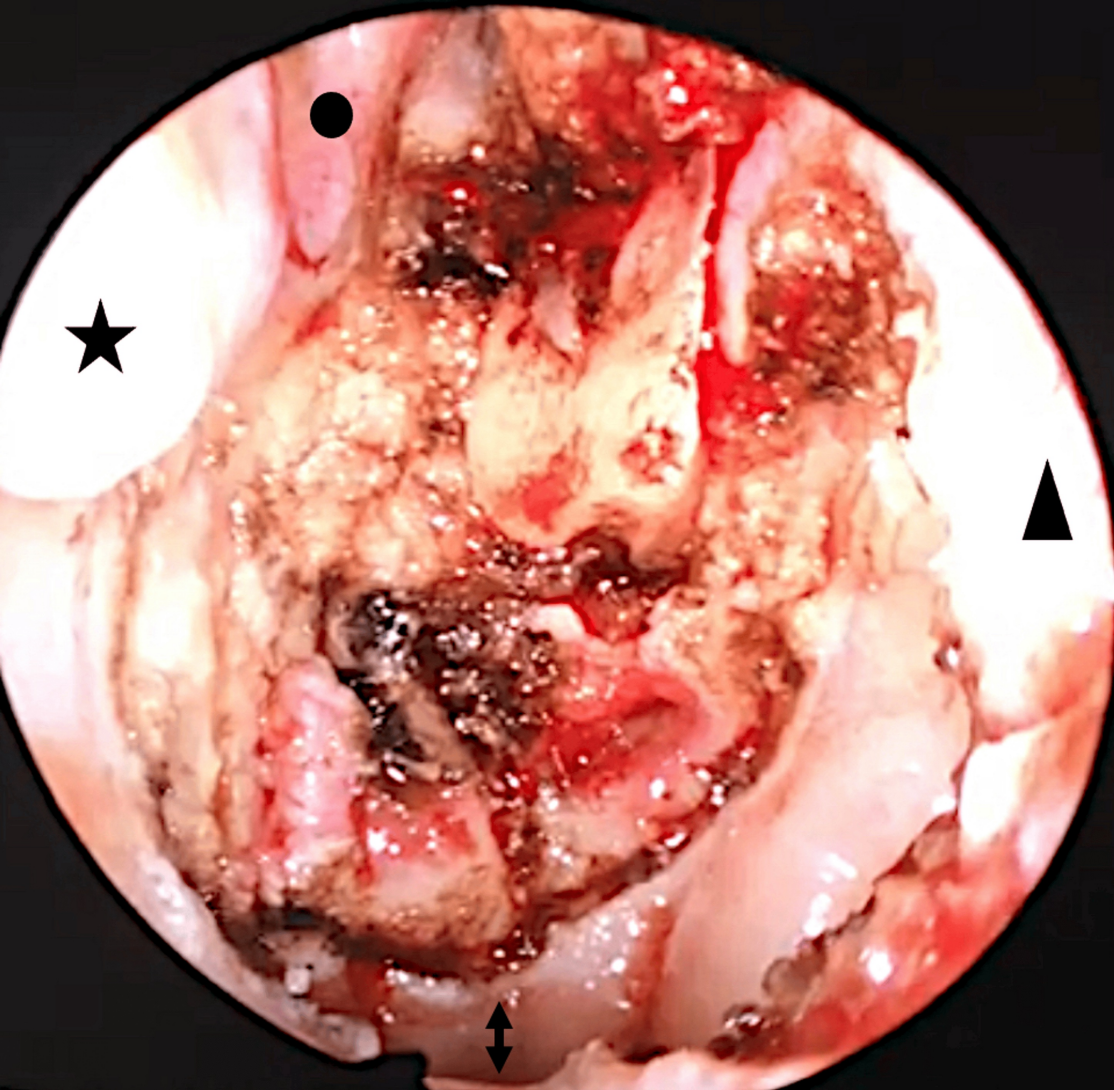
Nasopharyngoscopy after right endoscopic excision The black star, circle, triangle, cranial, and caudal head of the double-head arrow indicated the posterior attachment of the inferior and middle turbinate with no abnormalities, nasal septum, caudal nasopharyngeal resection line and nasal floor, respectively.

In July 2018, an MRI of the head and neck showed no abnormalities (Figure 1b). However, endoscopic examination of the nasopharynx revealed pigmented lesions in the right eustachian tube entrance and right nasopharyngeal roof. MDT recommended surgical treatment. In August 2018, en-block resection of these two lesions was performed by endonasal endoscopic surgery (Figure 2). Histology revealed metastatic melanoma, resected with clear margins.

Until the end of 2019, endoscopic follow-up examinations and MRI examinations of the head and neck revealed neither nasal nor nasopharyngeal tumor disease. The patient was then lost to follow-up. After unsuccessfully trying in 2021 to contact the patient, the patient’s contact persons informed us that he passed away in December 2020 of unknown cause.

Histologic examinations were carried out at an external pathology department other than that of our hospital. This resulted in the need for additional consent if we intended to obtain and publish the patient's histologic material, apart from the informed written consent that we used for this case report. However, this was no longer possible due to the patient’s decease and the absence of known relatives. Therefore, the external pathology department was not allowed to provide us with histologic figures or specific information from the histologic findings.

## Discussion

We performed an unsystematic data search in the PubMed database using possible combinations of the following keywords: “metastatic,” “metastasis,” "metastases,“ “paranasal,” “nasal,“ “maxillary,” “ethmoid,” “sphenoid,” “sinus,” “nasopharynx,” “cutaneous,” “malignant,” and “melanoma.” We evaluated human studies with abstracts in English. This research revealed 13 cases in 9 studies (from 1979 to 2019), two of which could not be retrieved and one of which was in Italian [4-12].

In the reports with complete data, the mean patient’s age was 41 years (range: 21-67 years). The mean time of metastasis after the initial diagnosis was 29 months (range: 6-72 months). Only surgically treated cases survived or were free of recurrence. Of the six cases treated surgically, five underwent adjuvant radiotherapy. For cases with complete data, the mean time of death after diagnosis of nasal metastasis was 22 months (range: 2-84 months). No recurrence was noted for only three patients treated surgically with adjuvant radiotherapy (12, 36, and 84 months; Table 1).

**Table 1:** Characteristics of the known well-described 13 cases of nasal/paranasal sinuses malignant melanoma metastasis from cutaneous malignant melanoma *Years; †w: woman; ‡m: man; §chemotherapy; ¶chemoradiotherapy; **after diagnosis of cutaneous melanoma; ††maxillary sinus; ‡‡ethmoid sinus; §§nasal cavity; ¶¶sphenoid sinus; ***all unilateral paranasal sinuses; †††multimodal treatment; ‡‡‡endoscopic sinus surgery; §§§radiotherapy; ¶¶¶chemotherapy/immunotherapy; ****in months; ††††after treatment of metastatic nasal disease in months. N/A: absent information due to non-retrieved paper.

Case	Date	Age/gender (y*)	Cutaneous melanoma site	Cutaneous melanoma treatment	Metastasis symptoms	Metastasis months**	Metastasis site	Metastasis MDT^†††^	Metastasis surgery	Metastasis RT^§§§^	Metastasis CT/IT^¶¶¶^	Metastasis recurrence****	Survival	Follow-up^††††^
1	1979 [4]	21/w^†^	Lumbar	Surgery	Epistaxis	15	MS^††^	Yes	Caldwell-Luc	Yes	No	2	No	4
2	1986 [5]	41/w^†^	Upper back	Surgery/CT^§^	Obstruction, pain, epistaxis	36	ES^‡‡^	Yes	External	Yes	No	No	Yes	12
3-5	1986 [6]	N/A	N/A	N/A	Epistaxis, pain	N/A	N/A	N/A	N/A	N/A	N/A	N/A	No	N/A
6	1989 [7]	N/A	N/A	N/A	N/A	N/A	MS^††^	Yes	Yes	Yes	No	No	Yes	N/A
7	1991 [8]	N/A	N/A	N/A	N/A	16	N/A	No	No	No	No	N/A	No	16
8	1995 [9]	30/m^‡^	Trunk	Surgery	N/A	41	NC^§§^	Yes	No	Yes	Yes	No	No	2
9	1995 [9]	62/m^‡^	Neck	Surgery	N/A	72	NC^§§^	No	No	No	Yes	No	No	7
10	2014 [10]	38/m^‡^	N/A	N/A	N/A	N/A	SS^¶¶^	Yes	ESS^‡‡‡^	Yes	No	36	No	36
11	2018 [11]	31/m^‡^	Thigh	CRTH^¶^	Obstruction	15	PS***	Yes	ESS^‡‡‡^	Yes	No	N/A	Yes	84
12	2019 [12]	67/m^‡^	Planar foot	CRTH^¶^	Epistaxis	6	MS^††^	No	No	No	Yes	N/A	No	13
13	2022 – current study	70/m^‡^	Big toe	Surgery	Obstruction, pain, epistaxis	112	NC^§§^	No	ESS^‡‡‡^	No	No	No	No	24

Moreover, 12 cases of cutaneous malignant melanoma metastasizing to the nasopharynx have been reported [6,9,13]. Epistaxis was the most frequent symptom. All nine patients described by Henderson et al. passed away from uncontrolled disseminated disease, regardless of the treatment modality (only one patient received immunotherapy) [6]. In their series, Billings et al. reported a 31-year-old man treated with chemotherapy 60 months after surgical treatment of a cutaneous melanoma of the trunk. The patient passed away from the disease 151 months after primary treatment. The second patient, a 46-year-old woman, passed away shortly after surgical treatment of metastatic melanoma, 41 months after surgical treatment of a cutaneous melanoma of the trunk [9]. Ramamurthy et al. reported a 37-year-old man with melanoma metastases of the nasopharynx and the right tonsil 24 months after surgical treatment of cutaneous melanoma of the right shoulder. The patient was referred for palliative treatment [13].

In the case described here, we reported the latest (approximately 10 years later) occurrence of metastasis after cutaneous melanoma (Table 1). Moreover, we noted two metachronous metastatic sites of the left infundibulum and the right nasopharynx. Additionally, we reported a 24-month disease-free survival (before the patient passed away for unknown reasons) with monotherapy, i.e., endonasal endoscopic surgery, while the other three out of 14 cases that survived had multimodal treatment, i.e., surgery with adjuvant radiotherapy. Obviously, these data do not justify monotherapy, i.e., surgery, as the only treatment option.

Data described by Henderson et al. [6] and Billings et al. [9] might somehow indicate the incidence of metastasis of cutaneous melanoma to the nose and nasopharynx, respectively. Henderson et al. noted 3 and 9 patients with melanoma metastasis of the nose and the nasopharynx, respectively, out of 8,823 patients with cutaneous melanoma at their institution [6]. Similarly, Billings et al. identified two patients with nasal- and two patients with nasopharyngeal melanoma metastasis in the pathology records at their institution from 1963 to 1993 [9]. Similar research in multiple centers worldwide might reveal more cases of nasal or nasopharyngeal melanoma metastases. Therefore, the total number of cases might be currently underestimated. Autopsy studies have revealed an incidence of metastases to the head and neck in 2% to 9.3% of those with cutaneous melanoma [14-16].

Molecular changes in the genes BRAF, NRAS, PI3K-ATK/PTEN, p53, CDK4/CDKN2A, c-KIT, MC1R, and cadherin are considered to be associated with metastatic malignant melanoma [17]. However, Luan et al. mentioned CDK1, FOXM1, KIF11, and RFC4 as the most significant for metastatic potential. Specifically, the UALCAN database suggested that CDK1 was significantly upregulated in metastatic melanoma compared with primary melanoma and that high expression of CDK1 was positively correlated with poor prognosis. Moreover, CDK1 plays an important role in the microenvironment of metastatic melanoma by regulating the tumor infiltration of immune cells [18].

The differentiation between primary mucosal melanoma and metastatic cutaneous melanoma is crucial since it might affect treatment strategy. Primary mucosal melanoma might necessitate a more aggressive surgical resection in contrast to melanoma metastasis, which might occasionally need a more palliative approach. Billings et al. described some pathologic features, which favor metastatic melanoma presence [9]. The most constant characteristic is intact overlying mucosa [19]. Additional features include lack of junctional activity, epidermal migration, and lack of pigmentation (Table 2) [9].

**Table 2:** Distinct pathologic features that can differentiate primary and metastatic melanoma lesions *According to Billings et al. [9].

Presence of pathologic feature*	Primary melanoma*	Metastatic melanoma*
Junctional activity	44%	0%
Epidermal migration	38%	0%
Pigmentation	69%	20%
Extension of melanotic pigment into minor salivary glands	25%	0%

Metastases of the above-mentioned cases were found in the head and neck. This location might not be in line with a direct spread from the primary focus since there was neither lung involvement nor documented open foramen ovale. This might lie in the vertebral venous system [20]. After cadaver injections, Batson described an additional venous system, i.e., the vertebral venous system, apart from the already-known cava, pulmonary, and portal venous systems. It is composed of veins of the brain, skull, neck, viscera, vertebral column, and body-wall veins. The vertebral venous system was considered a separate, although overlapping, system of veins, which might explain these atypical metastatic routes [20].

Some characteristics at the time of the diagnosis of cutaneous melanoma are worth mentioning. Interestingly, only four cases mentioned lymph node involvement at that time [4,11-13]. Only one study reported ulceration of the cutaneous melanoma lesion [11]. Moreover, only four studies stated the Clark invasion stage, which was II, III [9], III [5], and V [4]. Furthermore, only two studies reported the Breslow thickness (0.9 and 3.0 mm) [9] and histologic cutaneous melanoma type (nodular [5] and amelanotic [11]). Lastly, no study mentioned the BRAF mutation type.

This article has some limitations that are worth mentioning. Treatment of metastatic melanoma has greatly advanced in recent years. Most of the cases in Table 1 are quite old. Therefore, it is important to interpret Table 1 carefully. Most importantly, the absence of documentation of the examined molecular markers, such as BRAF, c-KIT, and CDK1, should not disorient readers from the importance of immunotherapeutic agents in metastatic melanoma.

## Conclusions

This report described a patient with two surgically treated metachronous metastases of the nose and nasopharynx after diagnosis of cutaneous melanoma. The histopathologic differentiation between primary mucosal and metastatic cutaneous melanoma remains critical. Adequate surgical treatment is crucial. More data are needed to further understand the role of adjuvant radiotherapy.
